# Common Bile Duct Access After Bariatric Surgery: A Narrative Review of Endoscopic Considerations for Treatment of Choledocholithiasis

**DOI:** 10.3390/diagnostics16132042

**Published:** 2026-06-30

**Authors:** Anna Booras, Jessica Zaman, Vladimir Davidyuk, Sandra DiBrito

**Affiliations:** Albany Medical Center, Department of Surgery, 43 New Scotland Avenue, Albany, NY 12208, USA

**Keywords:** choledocholithiasis, bariatric surgery, Roux-en-Y gastric bypass, transgastric ERCP, common bile duct exploration, EDGE, cholangioscopy

## Abstract

Choledocholithiasis after bariatric surgery presents a unique set of challenges for surgeons and gastroenterologists. This narrative review discusses the most common procedural interventions for choledocholithiasis in patients after commonly performed bariatric procedures, including laparoscopic transgastric ERCP, rendezvous ERCP, laparoscopic common bile duct exploration, cholangioscopy, and the EDGE procedure. All interventions maintain a technical and clinical success rate greater than 80%, but adverse outcome rates can vary from 8 to 22% and length of stay ranges from 1 to 17 days post procedure. Operative times range from 100 to 350 min. There are additional considerations, including procedure complexity, resource utilization, and limited availability depending on hospital system. Given the limited published literature available, there remains no commonly accepted algorithm for guiding which procedure to pursue. This review discusses considerations and reviews the current literature to offer suggestions for guidance for procedure choice in treating post-bariatric surgery patients with choledocholithiasis.

## 1. Introduction

Obesity remains a disease of epidemic proportions. As of 2023, 40.3% of American adults met criteria for obesity (BMI ≥ 30 kg/m^2^), with 9.4% of Americans meeting criteria for severe obesity (BMI ≥ 40 kg/m^2^) [1]. Bariatric surgery is a popular intervention with long-standing success in resolution of obesity [2]. However, post-bariatric surgery patients can present a unique set of complications in the gastrointestinal tract. Choledocholithiasis is a challenging disease pathology to treat in patients following bariatric surgery due to altered intraabdominal anatomy. Traditional use of standard endoscopic retrograde cholangiopancreatography is typically not feasible in patients who have undergone Roux-en-Y gastric bypass. As bariatric surgery patients with significant weight loss are at increased likelihood of developing gallstones and associated pathology, this presents a clinical challenge with increasing incidence. Interventions, including transgastric endoscopic retrograde cholangiopancreatography and minimally invasive common bile duct exploration, are the standard of care for many patients post-bariatric surgery with choledocholithiasis. Additional technological advancements in the field of cholangioscopy and advanced endoscopy are making progress in improving care for these patients but are not yet mainstream. In this narrative review, we present the pathophysiology of the disease, anatomical considerations in post-bariatric anatomy, technical considerations, and outcomes of the various procedures to manage choledocholithiasis in the bariatric patient.

## 2. Methods

A structured literature search was conducted using PubMed to identify studies relevant to transgastric ERCPs and choledocholithiasis in bariatric surgery patients. The review focused on the use of transgastric ERCP as an approach for biliary and pancreatic duct access, particularly in patients with surgically altered anatomy for bariatric purposes.

The PubMed database was searched using combinations of the following keywords and MeSH terms: “Cholangiopancreatography”, “Gastric Bypass”, “Roux-en-Y, ” “Bariatric Surgery”, “Endoscopy”, “Gastrostomy”, “Choledocholithiasis”, “Common Bile Duct Diseases”, “Biliary Tract Diseases”, “Obstructive Jaundice”, “transgastric ERCP”, “trans-gastric ERCP, "laparoscopic-assisted ERCP, ” “laparoscopic transgastric ERCP”, “EDGE procedure” and “biliary access after bariatric surgery.” Boolean operators were used to broaden and refine the search strategy, including combinations.

Articles were screened based on title and abstract for relevance to transgastric ERCP techniques, indications, outcomes, adverse events, technical success, clinical success, and comparisons with alternative approaches. Full-text articles were reviewed when abstracts suggested direct relevance to the topic. Studies were included if they discussed transgastric ERCPs in adult patients, described procedural technique or clinical outcomes, or compared transgastric ERCP with other methods of biliary access in altered gastrointestinal anatomy.

Exclusion criteria included articles not available in English, studies and reports that discussed standard ERCP without addressing transgastric or altered-anatomy approaches. Case reports, case series, retrospective cohort studies, systematic reviews, and comparative studies were considered because of the relatively specialized nature of the procedure and the limited number of large prospective trials available.

Data were extracted qualitatively from selected articles and organized according to procedural approach, patient population, indication for ERCP, technical success, clinical success, complications, need for repeat intervention, and comparison with alternative techniques. The findings were then synthesized narratively to summarize current evidence, identify trends in outcomes and safety, and highlight limitations in the existing literature.

## 3. Pathophysiology of Choledocholithiasis

Choledocholithiasis is the presence of one or more stones in the common bile duct (CBD) after stones form in the gallbladder and then migrate into the CBD or form primarily within the duct, which is significantly less common. Gallstones form when bile components, most often cholesterol or pigment, precipitate out of solution and progressively aggregate into calculi [3,4].

The root of the pathophysiology of choledocholithiasis is mechanical obstruction of bile flow. When a stone lodges in the common bile duct, bile can no longer drain from the liver into the duodenum. This raises intraductal pressure and causes upstream dilation of the biliary tree. Stagnant bile and ductal distention irritate the biliary epithelium and impair normal hepatic excretion of bilirubin and bile salts [5].

If the obstruction persists, the stagnant bile creates an environment that favors bacterial ascent from the duodenum, which can lead to ductal inflammation and infection with cholangitis. If the stone obstructs near the ampulla and blocks pancreatic duct drainage, the pressure gradient increases in the pancreatic ductal system and pancreatic enzymes may additionally become activated, causing gallstone pancreatitis. These are major significant downstream consequences of choledocholithiasis that can result in life-threatening illness [6].

Recent weight loss, particularly when rapid, is a recognized risk factor for cholelithiasis and related pathologies, particularly choledocholithiasis, because it promotes gallstone formation and subsequent migration of stones into the common bile duct. Rapid mobilization of adipose stores increases hepatic cholesterol secretion, resulting in bile that is supersaturated with cholesterol, which promotes stone formation. In addition, markedly reduced caloric intake and decreased dietary fat consumption impairs normal gallbladder contraction, leading to bile stasis and incomplete gallbladder emptying. The combination of cholesterol-supersaturated bile and gallbladder hypomotility facilitates the development of cholelithiasis, and subsequent migration of stones into the common bile duct results in choledocholithiasis. This is particularly relevant in patients undergoing bariatric surgery or other forms of rapid weight reduction [7].

Once choledocholithiasis is diagnosed, the most common first-line treatment is to proceed with endoscopic retrograde cholangiopancreatography (ERCP). In this technique, an endoscope is used to gain access to the biliary tree through the ampulla of Vater, cannulate the bile duct, and extract the stone(s) with a balloon or basket, and often perform a sphincterotomy. More advanced techniques, including CBD balloon dilation of strictures or mechanical lithotripsy of stones, can also be facilitated by endoscopic access with ERCP if necessary to relieve biliary obstruction. A cholecystectomy will often be pursued in a subsequent procedure to prevent recurrent obstruction. If the patient is undergoing cholecystectomy at the time when a duct stone is identified, a laparoscopic common bile duct exploration may be performed to remove the stone and relieve biliary obstruction [8].

## 4. Weight Loss Procedures and Anatomic Considerations

### 4.1. Sleeve Gastrectomy

**Figure 1:**
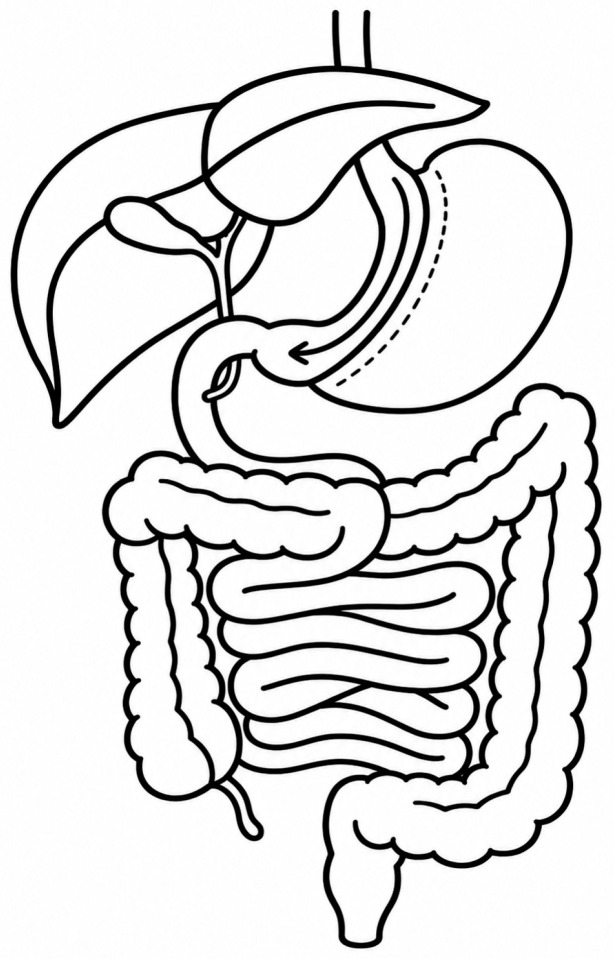
Diagram of altered anatomy after sleeve gastrectomy [11]. Arrow demonstrates flow of enteric contents.

A sleeve gastrectomy is a bariatric surgical procedure involving longitudinal resection of the greater curvature of the stomach, creating a narrow, tubularized stomach, maintaining gastric blood flow along the lesser curvature [Figure 1]. By significantly reducing gastric volume, the procedure promotes weight loss through restriction of oral intake. In addition, there are multiple alterations in hormonal signaling which impact appetite regulation, particularly through decreased ghrelin production [9] and increases in glucagon like peptide 1 (GLP 1) secretion and peptide YY (PYY) secretion due to accelerated gastric emptying and alterations in the bile–gut axis [10].

### 4.2. Gastric Banding

**Figure 2:**
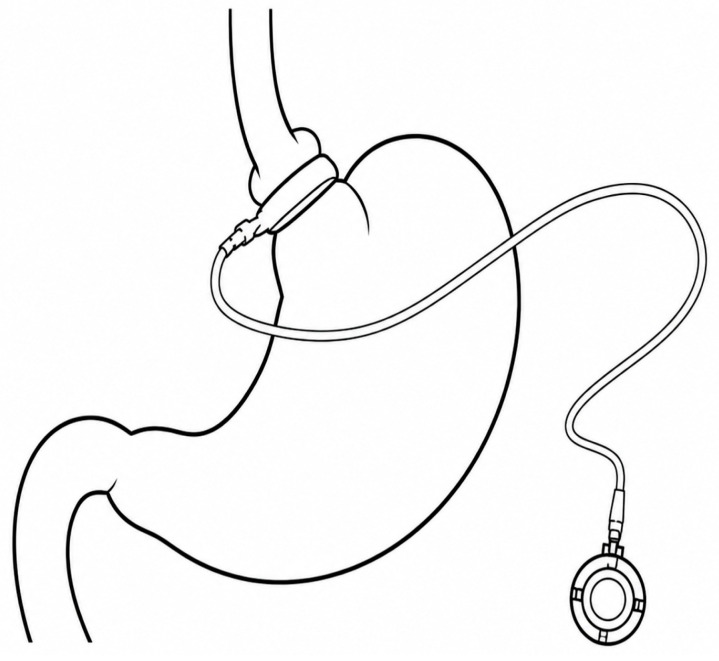
Diagram of altered anatomy after gastric banding [13].

Gastric banding is a restrictive bariatric procedure in which an adjustable silicone band is laparoscopically placed around the proximal stomach, just below the gastroesophageal junction [Figure 2]. The band creates a small gastric pouch and a narrowed outlet, limiting the amount of food that can be consumed at one time and promoting early satiety. The band is connected to a subcutaneous access port, allowing postoperative adjustment of band tightness by adding or removing saline. Unlike other weight loss surgical procedures, gastric banding does not involve intestinal rerouting or gastric resection [2]. It thereby has limited impact on gut hormone secretion and weight regulation. As a result, adjustable gastric banding has meager utility for durable weight loss and higher risk of complications due to concerns for band slippage or erosion [12].

### 4.3. Duodenal Switch

**Figure 3:**
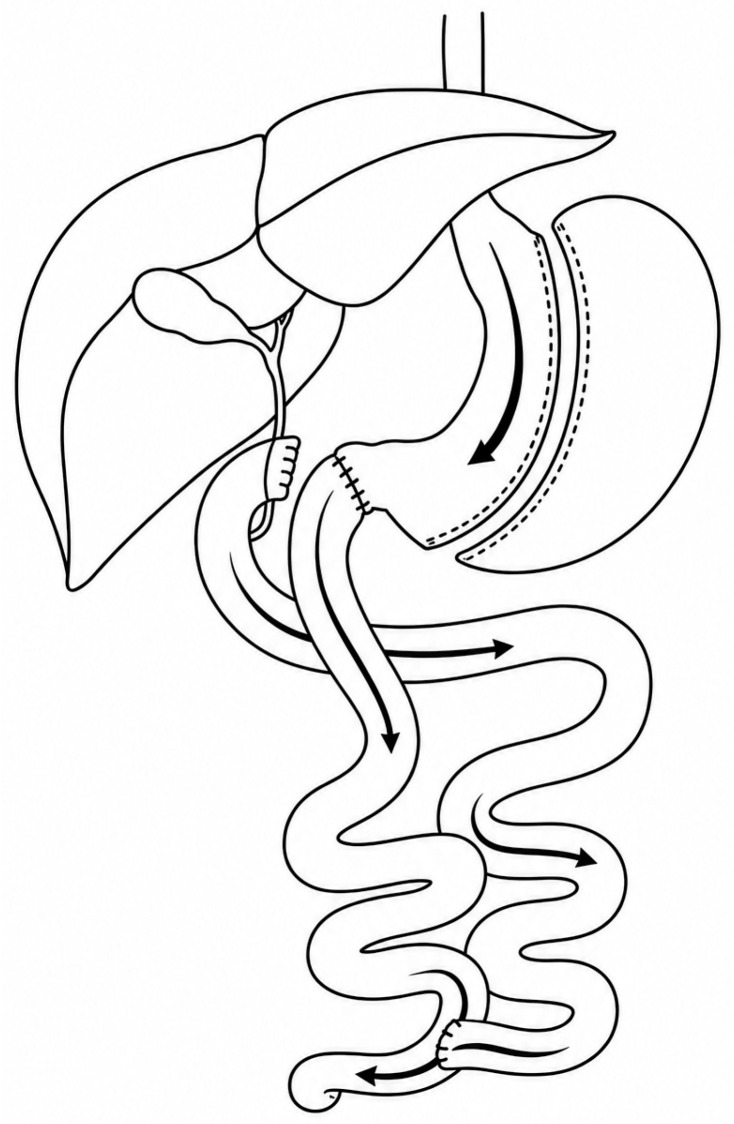
Diagram of altered anatomy after duodenal switch surgery [16]. Arrow demonstrates flow of enteric contents.

Duodenal switch is a bariatric surgical procedure that combines restrictive and malabsorptive weight-loss mechanisms. It typically begins with a sleeve gastrectomy, in which a large portion of the stomach is removed to create a smaller tubular stomach [Figure 3]. The small intestine is then divided near the duodenum and rerouted in the distal ileum (typically 100 cm proximal to the ileocecal valve) so that food bypasses a significant portion of the small bowel, reducing calorie and nutrient absorption. This procedure preserves the pylorus, which may help regulate gastric emptying, but it requires lifelong nutritional monitoring and supplementation due to the risk of vitamin, mineral, and protein deficiencies [14]. The duodenal switch procedure has been shown to be highly effective for weight loss but is infrequently utilized due to concern for major complications with long-term nutritional deficiencies and protein malnutrition [15].

### 4.4. Roux-en-Y Gastric Bypass: Anatomic Considerations and Bile Duct Access

**Figure 4:**
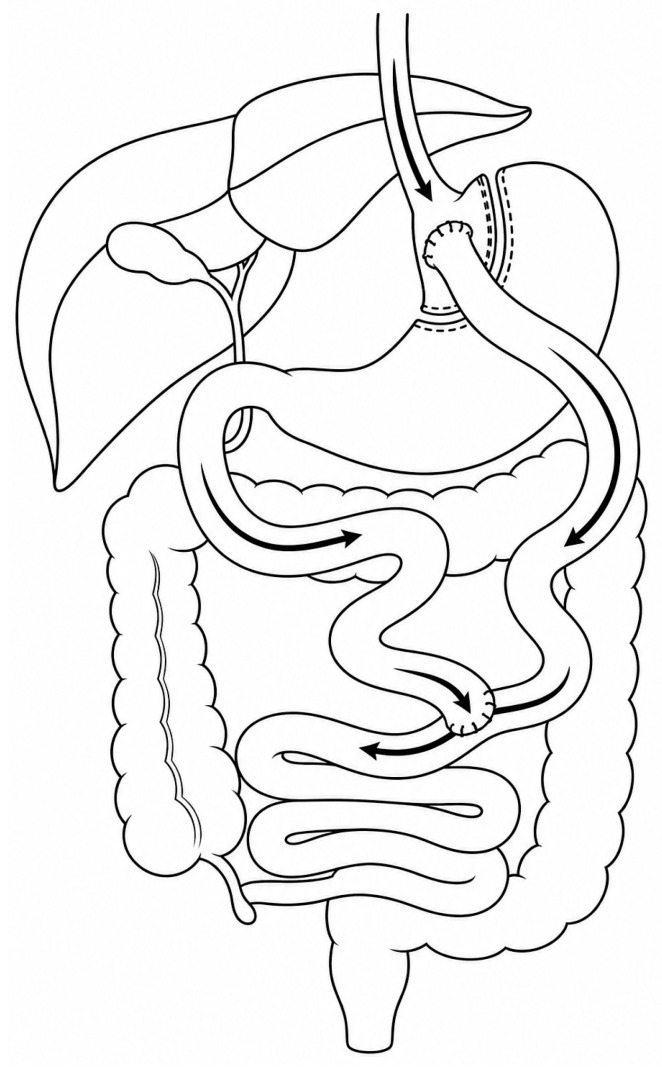
Diagram of altered anatomy after RNYGB surgery [17]. Arrow demonstrates flow of enteric contents.

In a Roux-en-Y gastric bypass (RNYGB), a small gastric pouch is created from the proximal stomach and anastomosed to a limb of jejunum. “Roux-en-Y” refers to a surgically altered gastrointestinal tract that creates a “Y” shape configuration. Specifically in gastric bypass procedures, the continuity between the gastric pouch, duodenum, and proximal jejunum is divided and reconstructed to create separate alimentary (roux) and biliopancreatic limbs [Figure 4]. This altered anatomy bypasses the majority of the stomach, the entire duodenum, and a variable length of proximal jejunum from the alimentary stream (typically 125–150 cm roux limb) [2].

RNYGB promotes weight loss through both restriction of oral intake and partial malabsorption, while also producing favorable hormonal and metabolic effects that can improve obesity-related comorbidities such as type 2 diabetes, hypertension, and gastroesophageal reflux disease. Patients often lose 60% of excess weight within 6 months and about 77% of excess weight by 12 months. On average, many patients still maintain around 50% of their excess weight loss at 5 years [16]. The rapid weight loss following RNYGB makes these patients more susceptible to gallstones and their associated complications such as choledocholithiasis [18,19].

Accessing the CBD is a major concern with the RNYGB procedure when compared to previously described bariatric surgery procedures. Due to the anatomical configuration of a Roux-en-Y gastric bypass, patients with choledocholithiasis are often unable to undergo standard ERCPs for treatment, as the duodenoscope can no longer reach the ampulla to cannulate the CBD. The laparoscopic or robotic-assisted transgastric endoscopic retrograde cholangiopancreatography has become the mainstay treatment option to manage acute biliary obstruction in patients with a history of RNYGB [20].

There is limited published data on the frequency and technical aspects of the transgastric ERCP procedure. The most recent meta-analysis was published in 2019 and featured only 931 transgastric-ERCP cases from 13 studies that occurred over the course of 8 years [21]. Similarly, the vast majority of these studies only examined the laparoscopic or open approach of the transgastric ERCP, with robotic-assisted transgastric ERCPs unrepresented in the literature [19,20,21]. Literature search of specifically robotic-assisted transgastric ERCPs yields only one study that published outcomes for two cases [22]. This data is immensely limited, as no comparative trials exist. Additionally, newer technology, such as the Spyglass cholangioscopy that offers an alternative treatment mechanism for choledocholithiasis in post-bariatric surgery patients, has not been explored.

It is presently unclear which approach is best used in this population and what options exist for clinicians with access to the myriad of technologies that are being developed in real time. We aim to describe the scope of the problem and an evidence-based discussion of the existing options to guide clinicians in their decision-making around this complex patient population.

## 5. Procedural Considerations for Bile Duct Access

### 5.1. Fluoroscopy

Fluoroscopy is an adjunct for both operative and endoscopic interventions on the cystic duct and common bile duct. Fluoroscopy-assisted operative procedures are performed with the aid of real-time X-ray imaging to guide instrument placement, confirm anatomy, and improve procedural accuracy. Fluoroscopy allows the operator to visualize internal structures dynamically during a procedure. This imaging guidance can help localize pathology, facilitate access to target structures, confirm successful intervention, and reduce the need for more extensive dissection in open operations. In operative settings, fluoroscopy is often used to enhance precision and safety while supporting minimally invasive approaches [23].

### 5.2. Endoscopic Retrograde Cholangiopancreatography

Endoscopic retrograde cholangiopancreatography (ERCP) is a combined endoscopic and fluoroscopic procedure used to diagnose and treat disorders of the biliary and pancreatic ductal systems. It is typically performed under monitored anesthesia care or general anesthesia with the patient in the prone or semi-prone position. A duodenoscope is advanced through the mouth, esophagus, and stomach into the second portion of the duodenum, where the major papilla is identified [24].

A duodenoscope is a flexible, side-viewing endoscope designed for use in ERCPs and other procedures involving the duodenum, ampulla, and pancreatobiliary system. Unlike a standard forward-viewing upper endoscope, the duodenoscope has a laterally oriented optical lens that facilitates visualization of the major papilla when the instrument is positioned in the second portion of the duodenum [24].

It also contains an elevator mechanism at the distal tip, which allows the endoscopist to precisely control accessories such as cannulas, sphincterotomes, guidewires, balloons, and stents during selective duct cannulation and therapeutic intervention. Its design permits stable positioning in front of the papilla and allows fine manipulation of endoscopic accessories within the biliary and pancreatic ducts under fluoroscopic guidance [24].

**Figure 5:**
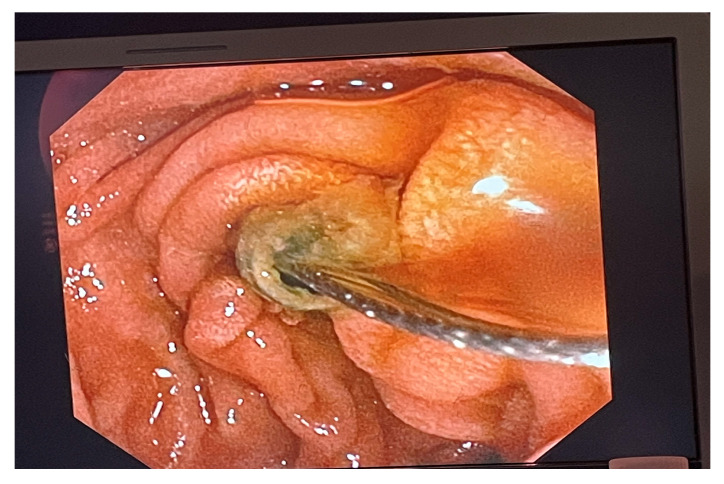
Wire from duodenoscope cannulating ampulla of common bile duct (Attending Surgeon: Vladimir Davidyuk, MD).

Once the papilla is brought into view, a cannulating catheter or sphincterotome is used to selectively access the common bile duct or pancreatic duct [Figure 5]. Contrast is then injected under fluoroscopy to define the ductal anatomy and identify pathology such as choledocholithiasis, biliary stricture, obstruction, leak, or pancreatic duct abnormalities. After ductal cannulation, therapeutic interventions may be performed as indicated, including biliary or pancreatic sphincterotomy, balloon sweeping, stone extraction, dilation of strictures, brush cytology or biopsy, and placement or exchange of stents. Post-procedural complications include pancreatitis, bleeding, perforation, or cholangitis [24].

Transoral ERCP is a cornerstone in the management of pancreaticobiliary disease, as it allows direct diagnostic and therapeutic intervention using standard duodenoscopic access to the ampulla. In patients with normal upper gastrointestinal anatomy, it provides an effective and minimally invasive approach for the evaluation and treatment of biliary and pancreatic duct pathology.

### 5.3. Laparoscopic or Robotic-Assisted Transgastric Endoscopic Retrograde Cholangiopancreatography

In patients with RNYGB anatomy, the major papilla remains within the excluded duodenum and is no longer accessible by a conventional transoral route using a standard duodenoscope. This anatomic rearrangement has important implications for endoscopic access to the biliary and pancreatic systems and often necessitates alternative techniques, such as laparoscopic-assisted transgastric ERCP, balloon-assisted enteroscopy, or endoscopic ultrasound-directed transgastric access (EDGE procedure), when pancreatobiliary intervention is required. A laparoscopic or robotic-assisted transgastric ERCP provides access to the biliary tree by combining minimally invasive surgical entry into the excluded stomach with endoscopic cannulation of the ampulla [25].

Transgastric ERCP is performed under general anesthesia with the patient in the supine position. After laparoscopic access to the abdomen is established, the excluded gastric remnant is identified and brought into view, and a gastrotomy is created on the anterior wall. A large trocar or access port (typically 15 mm) is then placed directly into the gastric remnant and secured to allow passage of a standard duodenoscope. The endoscopist advances the duodenoscope through the gastrotomy port, across the pylorus, and into the second portion of the duodenum, where the major papilla is identified [25].

Once access is obtained, ERCP proceeds in a standard manner, including selective cannulation of the common bile duct or pancreatic duct, cholangiography or pancreatography, sphincterotomy if indicated, stone extraction, stent placement, or other therapeutic intervention as required by the underlying pathology. A rendezvous ERCP refers to a variation of the traditional ERCP, where the wire is passed antegrade through the cystic duct as opposed to retrograde through ampulla, either immediately following a cholecystostomy or creating a cystic ductotomy. This can also be facilitated with laparoscopic or robotic assistance if the endoscopist is unable to cannulate the ampulla in a standard retrograde fashion. After completion of the endoscopic intervention, the duodenoscope is removed, and the port in the gastrotomy is withdrawn. The gastrotomy is then closed and oversewn in a standard minimally invasive fashion, and the abdomen is irrigated and inspected for hemostasis before trocar removal and closure. If a plastic stent is placed into the biliary tree or pancreatic duct during transgastric ERCP, repeat procedure is necessary to remove the stent. In these cases, the gastrotomy site is not closed. Instead, a gastrostomy tube is placed in the remnant stomach at that site using a purse-string suture to close the gastrotomy around the tube, and the temporary gastrostomy is left in place in order to access the stomach again in a subsequent interval transgastric ERCP with stent removal. The gastrostomy tube will then be removed following the subsequent ERCP and the gastrotomy site is allowed to close [25].

Laparoscopic or robotic-assisted transgastric ERCP is particularly useful in post-RNYGB patients with choledocholithiasis, biliary obstruction, cholangitis, or other pancreaticobiliary pathology requiring intervention. It offers the advantage of using a conventional duodenoscope, thereby preserving familiar endoscopic orientation and allowing standard therapeutic maneuvers, while overcoming the altered anatomy that prevents traditional ERCP access.

### 5.4. Laparoscopic-Assisted Common Bile Duct Exploration

Laparoscopic-assisted common bile duct exploration (LCBDE) describes a minimally invasive surgical approach for the management of choledocholithiasis, often utilized if the patient is planned to undergo cholecystectomy at the time of procedure. The technique permits direct intraoperative assessment of the biliary system and facilitates ductal clearance using cholangiography and stone extraction devices introduced either via the cystic duct or through a choledochotomy. When performed in conjunction with laparoscopic cholecystectomy, it provides a single-stage treatment strategy for patients with concomitant gallbladder and common bile duct stones. Altered RNYGB anatomy does not preclude performance of an LCBDE. The contemporary literature supports this approach as a safe and effective alternative to staged ERCP-based management in appropriately selected patients, with the added benefits of definitive stone clearance during one anesthetic episode and the potential for shorter hospitalization and lower overall resource utilization [26]. Typical operative time can range from 125 to 190 min [27]. However, depending on the duration of symptoms, concurrent acute cholecystitis, and other patient factors, it may not be feasible or safe to proceed with CBD exploration if cholecystectomy is not also planned.

### 5.5. Cholangioscopy

Gradual innovations in cholangioscopy systems have allowed surgeons to access increasingly small ductal structures and perform interventions such as lithotripsy in settings where it had previously been impossible. The SpyGlass system (Boston Scientific, Marlborough, MA, USA) was first introduced in 2005 with a fiberoptic model and is now offering a digital iteration released in 2015 [28].

**Figure 6:**
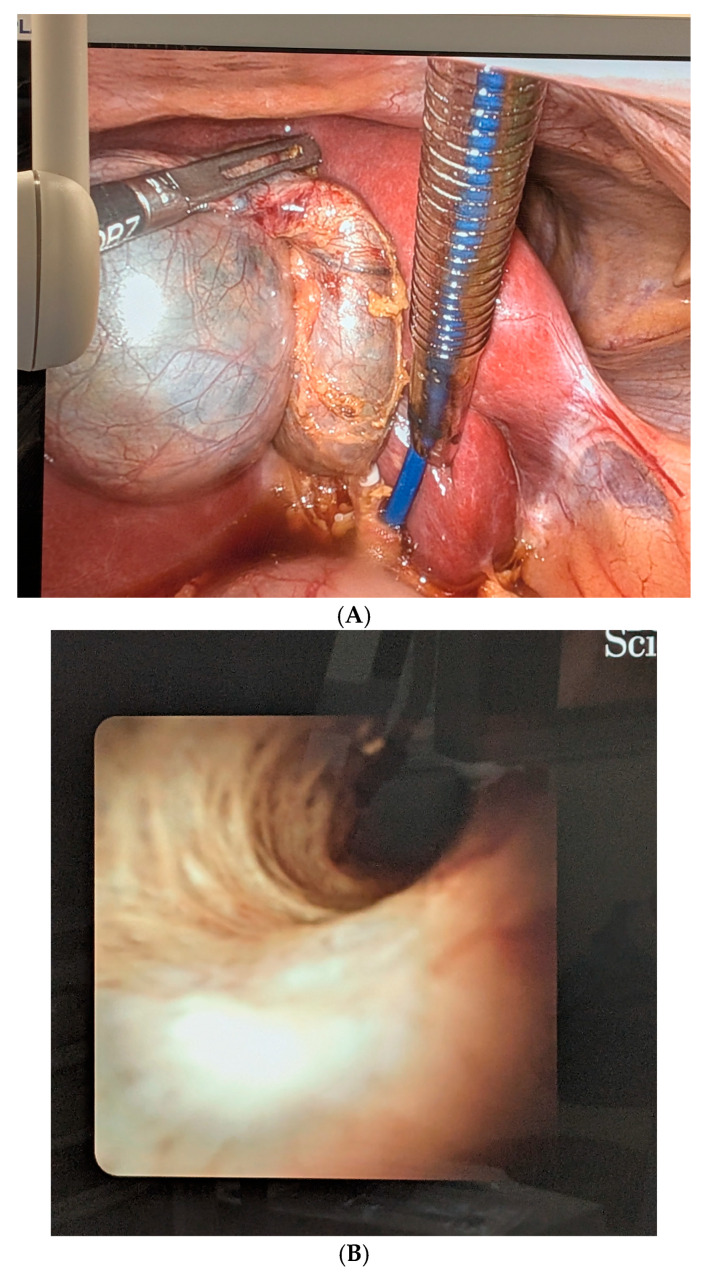
Intra-operative photos from SpyGlass use. (**A**) External view of ductotomy with scope placed intralumenal. (**B)** Internal view from cholangioscopy of CBD. (Attending Surgeon: Vladimir Davidyuk, MD.)

The SpyGlass direct visualization system is a single-operator cholangioscopy platform used during CBD exploration to permit real-time endoscopic visualization of the biliary or pancreatic ductal system [Figure 6]. This can be performed during open surgery but is increasingly feasible during laparoscopic and robotic surgery to allow for minimally invasive access to smaller biliary systems. This technology is being adopted by large academic hospital centers due to its promise of optimizing and improving treatment of choledocholithiasis in complex patients [29].

During these cases, a cholangioscope is introduced in a percutaneous fashion into the abdomen, guided by laparoscopic or robotic access, and introduced into the biliary system, most often through the cut cystic duct stump after a cholecystectomy or through a choledochotomy. This allows for visual endoscopic assessment of the biliary system. It is most commonly employed when conventional ERCP is not available, not technically possible (for patients with duodenal or pyloric stricture for example), or is unsuccessful and can be used in patients with surgically altered anatomy or complex biliary pathology, such as post-RNYGB. The technique enables both diagnostic and therapeutic intervention, including direct inspection of ductal mucosa, biopsy of concerning tissue, and management of difficult stones or strictures under visual guidance [30,31].

While cholangioscopy is not particularly novel, the smaller-caliber digital systems offer some advantages over larger scopes, but access remains limited. In these settings, transgastric ERCPs are a reliable option for patients with history of RNYGB and choledocholithiasis.

## 6. Discussion

### 6.1. Management of Choledocholithiasis in Patients with Complex Upper Gastrointestinal Anatomy

The management of choledocholithiasis has evolved to include a range of endoscopic and minimally invasive approaches that can be tailored to patient anatomy, operative risk, and available institutional expertise. Here, we discuss the current literature available for treatment of choledocholithiasis specifically for patients with RNYGB anatomy. Of note, available research in this topic is limited to small-powered studies.

### 6.2. Laparoscopic Transgastric ERCP

Endoscopic techniques offer an effective and minimally invasive solution for choledocholithiasis, especially for patients who are poor surgical candidates or have difficult anatomy as opposed to proceeding immediately with surgery [32].

Additionally, ERCP is a useful tool for treating post-cholecystectomy complications. For bile leaks after cholecystectomy, ERCP is the first-line treatment. Small leaks may be managed with sphincterotomy alone, while more severe or complex leaks often need stents [32]. For post-operative benign biliary strictures, ERCP works well with a 97% stricture resolution rate but may require multiple sessions over time [33].

In a recent review of the management of choledocholithiasis after bariatric surgery, authors include laparoscopic-assisted ERCP, balloon-assisted enteroscopy ERCP, endoscopic ultrasound (EUS)-directed transgastric approaches, and percutaneous surgical management as viable options. They conclude that there is not one universally best solution for every patient, because the ideal treatment depends on the type of prior bariatric operation and the resources and skill set of providers available [34]. Additional research is warranted as this field is undergoing technological advancement that would benefit from more controlled studies.

A Swedish database study from 2021 demonstrated that common bile duct stones were significantly more common in patients with prior gastric bypass undergoing cholecystectomy than in the general population undergoing cholecystectomy [35]. Overall, 12.7% of patients with prior gastric bypass undergoing cholecystectomy had choledocholithiasis compared to 9.0% in the general cholecystectomy population. Stones were managed in a variety of ways in the RNYGB group, with 20% of patients managed expectantly, 3% undergoing transgastric ERCP, 5% undergoing laparoscopic biliary exploration with choledochotomy, 9% undergoing open common duct exploration with choledochotomy, and 22% managed with transcystic stone extraction. Postoperative complications were rare, with minor complications occurring only 16% of the time, and there was no reported statistically significant difference in outcome between the different management options, although the numbers in each group were limiting in terms of statistical power. Transgastric ERCP took the longest time in the operating room, with a median time of about 350 min. Open and laparoscopic choledochotomy had the longest hospital stays after surgery. The authors concluded that transgastric ERCPs are considered feasible and safe but are complicated by the need for an experienced endoscopist and tend to take longer to complete. Transcystic extraction is less invasive but can be difficult for large stones or with a narrow cystic duct. Laparoscopic choledochotomy can remove stones of many sizes and locations but is technically more challenging and carries risk of bile leak or delayed stricture [35].

A study published in 2025 explored 44 patients treated between 2014 and 2022 with LCBDE following gastric surgery [36]. In 38 of the 44 cases, surgeons also performed a concomitant cholecystectomy. Patients with history of RNYGB, Billroth II subtotal gastrectomy, total gastrectomy, and subtotal gastrectomy with Roux-en-Y reconstruction were included. Cholelithiasis was confirmed at the time of surgery in 38 patients (85%). Nearly all (97%) patients with confirmed stones had successful duct clearance and only 7% had a serious complication (as defined by Clavien-Dindo > 3a). The authors concluded that LCBDE appears to be a safe and highly effective option for patients with bile duct stones after previous gastric surgery [36].

Both percutaneous access transgastric ERCPs and common bile duct exploration are viable options for managing choledocholithiasis in patients with gastric bypass anatomy. A head-to-head comparison was performed in a study by Zaigham et al., studying LCBDE and laparoscopy-assisted transgastric endoscopic retrograde cholangiography [37]. The group used the Swedish national registry data from 2011 to 2020 and retrospectively identified 550 patients with bile duct stones found during gallbladder surgery after prior gastric bypass. A majority were treated with LCBDE and 26% were treated with transgastric ERCP. Both procedures had very low intraoperative complication rates (1% for LCBDE vs. 2% for transgastric ERCP), and postoperative complications within 30 days were also similar (16% for LCBDE vs. 18% for transgastric ERCPs). LCBDE was faster, with an operating time about 31 min shorter on average than transgastric ERCPs. Stones under 4 mm were more common in the LCBDE group. Transgastric ERCPs were used more often in complex situations, including an acute presentation and larger stones (over 8 mm). This retrospective cohort comparison has clear bias with selection of procedure type for each patient. However, this article does support the use of clinical decision making to select the most appropriate procedure for each patient to achieve the best outcome while limiting complications and operative time [37].

Patient-centered outcomes with regards to choledocholithiasis include resolution of jaundice, cholangitis, biliary colic, and pancreatitis. In a 2025 systematic review/meta-analysis of post-RNYGB ERCP techniques, laparoscopic transgastric ERCP had a pooled clinical success rate of 92% and technical success rate of 93% [38]. Another reported benefit was avoidance of more invasive surgery, such as open technique [39]. Reduced recovery burden, including postoperative pain and decreased hospital length of stay has been reported [40]. There has been a lack of published data on other effects on daily living following transgastric ERCP, such as mobility after surgery and return to work. One review published in 2024 did highlight negative effects on patient outcomes including increased wait time and frequent need for transfer to higher level of care outside tertiary centers [39]. Overall, patient satisfaction with laparoscopic transgastric ERCP tends to be high, with reported benefits of reliable likelihood of duct clearance and definitive therapy [38].

### 6.3. Rendezvous ERCP

An additional technique to consider for challenging intraoperative ERCP is the rendezvous procedure. This involves passing the wire antegrade through the common bile duct, either through choledochotomy or cystic duct orifice. A recent retrospective cohort study evaluated patients with history of RNYGB against patients who had no history of bariatric surgery, studying management of common bile duct stones with rendezvous ERCP [41]. Patients with history of RNYGB underwent laparoscopic cholecystectomy plus laparoscopy-assisted transgastric rendezvous ERCP. The control group had laparoscopic cholecystectomy plus rendezvous ERCP. Data was acquired from the Swedish national registry and included 70 patients with prior RNYGB and 4342 patients without prior upper abdominal surgery. CBD stones were found in 60 RNYGB patients and 3067 control patients. The reported therapeutic success rate was 100% in the RNYGB rendezvous ERCP group versus 91.4% in the non-RNYGB standard group. Complications during the operation were more common in the RNYGB group. Perioperative adverse events occurred significantly more frequently in the RNYGB patients (8.8%) compared to control (2.3%). Despite the higher complication rate, authors concluded that laparoscopic-assisted rendezvous ERCP performed during cholecystectomy is a viable single-session approach for clearing bile duct stones in RNYGB patients during cholecystectomy [41]. Another small case series examined laparoscopic-assisted rendezvous ERCP in four patients with prior RNYGB who had CBD stones [42]. In all four patients, the procedure was successful in clearing the bile duct stones. The postoperative courses were uneventful, and the patients had normal amylase levels, which suggests no chemical post-procedure pancreatitis, which is the most common post-procedural complication of ERCPs, reportedly upwards of 15% [43]. For comparison, laparoscopic transgastric ERCP post-procedure pancreatitis is approximately 7% (95% CI of 6–8%) [38]. The average procedure time for rendezvous ERCP was 105 min. The average hospital stay after the procedure was 2 days. This limited study suggests fewer complications and a shorter operative time than standard transgastric ERCP with passing of the wire retrograde through the CBD; however, it should be noted this is underpowered [42].

Rendezvous ERCP has been shown to be safe in small studies that are limited by low power [41,42]; however, there are concerns regarding the risk profile for choledochotomy compared to cystic duct stump access when patients do not have a concomitant cholecystectomy. It should also be noted that rendezvous ERCP access may be challenging using standard positioning for laparoscopic cholecystectomy and may require intraoperative repositioning for cannulation of the bile duct.

### 6.4. EDGE Procedure

**Figure 7:**
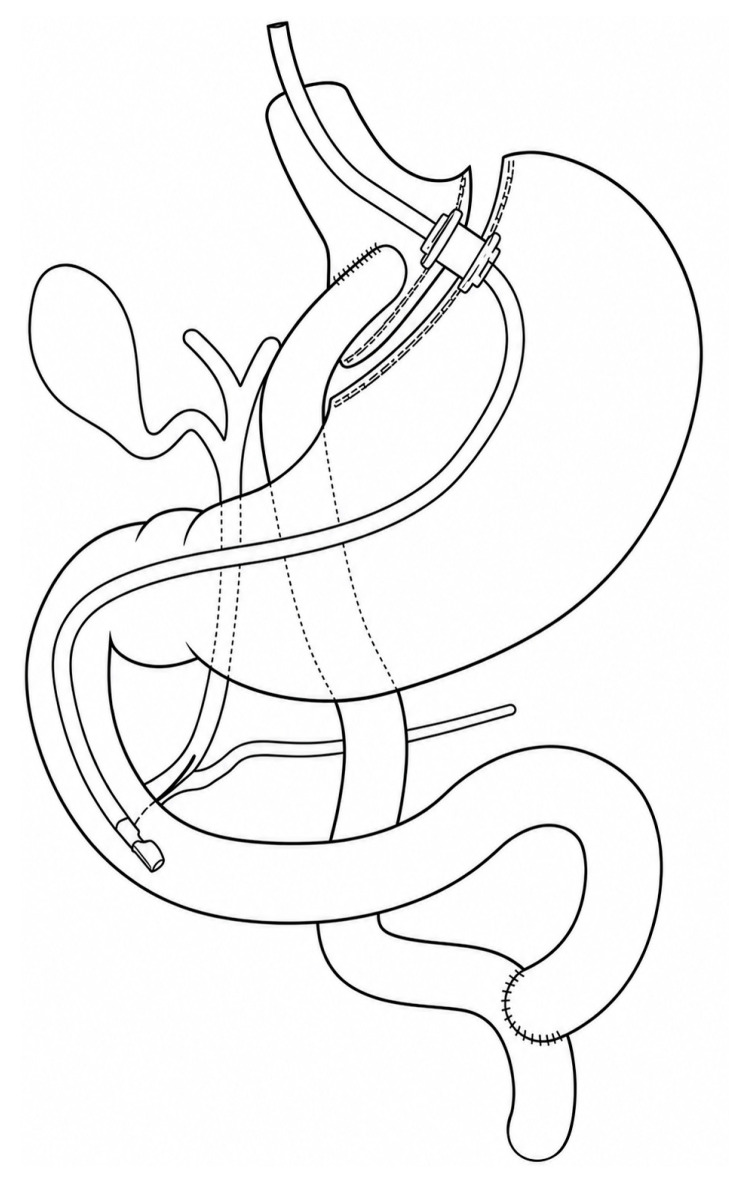
Diagram of EDGE procedure with LAMS stent [45]. Diagram shows scope intraluminal.

Another somewhat less popular approach to treating choledocholithiasis in patients with a history of gastric bypass includes the EUS-directed intraluminal transgastric ERCP or EDGE procedure that employs EUS guidance to place a 20 mm lumen-apposing metal stent (LAMS) to create access into the excluded stomach [44]. After a period of waiting while the gastro–gastric fistula tract is matured, in a separate procedure, a duodenoscope is passed through the stent to perform a conventional peroral ERCP [Figure 7].

A small retrospective study of the EDGE procedure in Canada identified only seven patients who underwent EDGE between 2019 and 2021 at tertiary centers in British Columbia and Quebec [44]. Choledocholithiasis was the indication for six patients and gallstone pancreatitis for one patient. Of note, none of the patients had ERCP during the same session as stent placement. The median time from LAMS placement to ERCP was 9 days, and the median time from ERCP to LAMS removal was 17 days. After ERCP, the fistula tract was actively managed to promote closure: five patients had the LAMS replaced with a double-pigtail stent, one patient had argon plasma coagulation, and one patient had the LAMS remaining in place while awaiting cholecystectomy at the time of study publication. In this cohort, 71% of cases required four separate procedures: LAMS placement, ERCP, LAMS removal with pigtail placement, and later pigtail removal. Success was high, with both technical and clinical success reported at 100%. No immediate or delayed adverse events were reported, but persistent fistula and unwanted post-procedural weight gain were noted as long-term negative effects. However, in one more challenging case, the excluded stomach could not be identified at first because of a hiatal hernia but, after repeat procedure, it was identified and LAMS was successful. The study is limited by low power, as the EGDE procedure is rarely performed. The study highlights that only 25% of surveyed Canadian centers with advanced endoscopic ultrasound services offer the EDGE procedure [44].

Overall adverse events of EDGE are reported to vary from 17 to 22% from a 2023 meta-analysis. This includes bleeding at 2–7%, persistent fistula of 1%, and procedure-associated perforation at 1–4%. Other complications include LAMS migration or dislodgement (15–16%), often the most common EDGE-specific issue. Post-ERCP pancreatitis has been published to be around 2%, but this was found to have no statistical significance when compared to laparoscopic transgastric ERCP [46].

Although EDGE is an attractive, minimally invasive option that is safe and efficacious in managing common duct stones for patients with RNYGB anatomy, its utility is hampered by the numerous critiques of the procedure. Primarily, the multi-step process is slow to resolve clinical issues, hospital stays are longer, it may not be as cost-effective as other options, and readmission rates are high [47].

### 6.5. Cholangioscopy Procedural Outcomes

Cholangioscopy allows for direct visualization inside the bile ducts and pancreatic ducts, as opposed to relying on fluoroscopic images during ERCP or standard cholangiogram. This can aid in both diagnosis and treatment, particularly when standard methods are limited. In cases with impacted, large, or challenging to reach stones, cholangioscopy in conjunction with additional technology can facilitate laser or electrohydraulic lithotripsy. In a multinational registry report, stone clearance after cholangioscopy procedures ranged from 82 to 97% [48].

Cholangioscopy may also add value in evaluation of biliary strictures. Direct endoscopic visualization can provide additional information missed with fluoroscopic evaluation. It is known that ERCP cytology and biopsy have limited sensitivity, only up to 59% combined in detecting malignancy [49]. Neovascularization, papillary projections, tortuous vessels and other suspicious features are visible and can increase sensitivity for detecting malignancy just by visual inspection [31]. Systematic reviews show that visually guided biopsy with single-operator cholangioscopy had about 60–69% sensitivity and 98% specificity for malignancy, significantly higher than for traditional ERCP. Visual impression alone was reported as 90% sensitive and 87% specific, though it is stressed that biopsy confirmation is still needed [31,50].

There is limited published literature directly comparing cholangioscopic exploration to ERCP. One study by Mohamed et al. explores Spyglass cholangioscopy and highlights some of the theorized benefits of Spyglass over other procedures to treat choledocholithiasis [51]. In this article, the authors stress that the index ERCP procedural failure is possible, with reported initial biliary duct clearance rates ranging from 72.5 to 80.8% at tertiary care centers [51].

To date, only one case study has investigated Spyglass cholangioscopy in the bariatric surgery population [52]. They presented a single case of a 45-year-old female patient with history of RNYGB who successfully underwent Spyglass cholangioscopy to treat intraductal calculi [52].

Each available procedure for treatment of choledocolithiasis offers varied success rates, adverse events, and lengths of stay. We provide an overview in Table 1 below [7,34,36,38,41,44,53,54,55,56].

### 6.6. Consideration of Cost and Resource Utilization

Another consideration for the aforementioned procedures is that these complex interventions often present logistical challenges for hospital coordination and carry significant costs. Coordination between the surgeon and advanced endoscopist can significantly delay cases, sometimes leading to longer hospital stays for patients and off-hour scheduling for cases [53,54,55].

The cost of these procedures widely depends on institution and insurance payer, with great variation due to hospital requirements and patient specifics [57]. A standard ERCP in a patient without surgically altered anatomy is typically in the order of thousands of dollars; however, a transgastric ERCP can cost 10-fold more. The estimated cost of a transgastric ERCP in the United States can range from $15,000 to 40,000. In complex patients with long hospital stays, the cost can exceed $50,000 if there are complications or repeat procedures. This is because it requires operating room access, as opposed to endoscopy suite, anesthesia, a multidisciplinary team, and subsequent inpatient observation. This is compared to the EDGE procedure, which is reported to initially cost approximately $9700 but also involves the cost of prolonged hospitalization and multiple procedures [58].

The cost of a laparoscopic common bile duct exploration with concurrent cholecystectomy costs approximately $36,000 due to operative time, cholangiography and fluoroscopy equipment use, and specific instrument use. LCBDE additionally is complicated by intraoperative coordination with fluoroscopy staff [59].

Streamlining the decision process for which is the optimal procedure to treat choledocholithiasis and the surgical anatomy-altered bariatric patient may help to eliminate the strain on resource utilization, multidisciplinary coordination and cost-effectiveness for these complex procedures.

## 7. Conclusions

**Figure 8:**
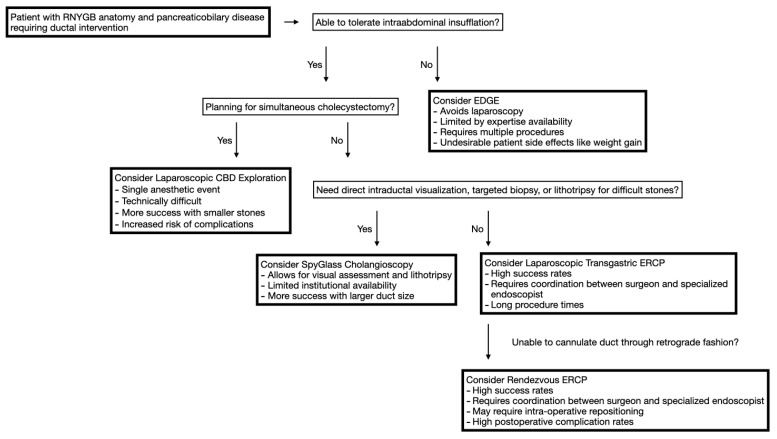
Proposed clinical decision-making algorithm for treatment of choledocholithiasis in patients after bariatric surgery.

The management of choledocholithiasis in patients with surgically altered anatomy, particularly after RNYGB, requires an individualized approach that balances technical feasibility, stone burden, patient stability, institutional expertise, and available resources. Although, we highlight that the available published research is extremely limited by small studies with low power and conclusions should be taken as exploratory considerations and not definitive, data-driven conclusions. Traditional ERCP remains a highly effective first-line option when normal anatomy permits access, but altered anatomy often necessitates alternative strategies, including laparoscopic-assisted transgastric ERCP, laparoscopic common bile duct exploration, rendezvous techniques, EDGE, and cholangioscopy-assisted interventions such as SpyGlass. The limited available literature suggests these techniques are safe and effective, but each carries distinct limitations, such as coordination between advanced endoscopy providers and surgeons, longer operative times, and specialized expertise. LCBDE provides durable stone clearance, but it is technically demanding and carries risks such as bile leak and stricture. EDGE is effective in selected centers but often requires multiple procedures and raises concerns about persistent gastro–gastric fistula and weight regain. Cholangioscopy expands therapeutic options, but its role in post-bariatric patients remains unexplored with robust research. Overall, current evidence supports a multidisciplinary, patient-specific treatment algorithm rather than a single preferred intervention [Figure 8].

## 8. Limitations

The scope of this narrative review is limited by available published research. No randomized control trial or prospective study exists examining laparoscopic transgastric ERCP and other approaches for patients with a history of bariatric surgery and choledocholithiasis. Additionally, existing studies are heterogenous and have small sample sizes, restricting the ability to calculate pooled effect sizes for a true meta-analysis. Virtually all studies examined in this review are retrospective, single-centered studies that may be subjected to selection bias.

## 9. Future Directions

Further research directly comparing techniques in a homogeneous patient population in prospective and randomized control trials would allow for creation of evidence-based guidelines in the approach to choledocholithiasis in populations with altered upper GI anatomy. Patients with choledocholithiasis in the setting of altered upper gastrointestinal anatomy, particularly bariatric surgery patients, are particularly challenging to manage.

### Outlook

Technological advancements will continue to positively impact the care of patients with choledocholithiasis and altered upper GI anatomy. Where traditional ERCP is not possible, opportunities to improve access to the biliary tree exist and current techniques should continue to be honed to minimize risk and maximize efficacy, while streamlining care that can often cause long length of stay.

## Figures and Tables

**Table 1:** Benefits and limitations of available treatment options for choledocholithiasis in post-RNGYB patients [7,34,36,38,41,44,53,54,55,56].

Procedure		Major Benefits	Major Limitations	Technical Success Rate	Clinical Success Rate	Adverse Event Rate	Number of Procedures	Length of Stay	Expertise Required
Transgastric ERCP	Laparo-scopic	Safe and efficient	Long procedure time (~350 min)	93%	92%	19%	1–2	2 days	Yes
	Robotic	Potential for reduced pain after surgery	Long procedure time (~163 min)	85%	90%	~8%	1–2	2 days	Yes
Rendevouz ERCP		Reduced rate of post-ERCP pancreatitis	Risk of bile leak	~100%	~100%	~8.8%	1–2	2 days	Yes
EDGE Procedure		Allows for traditional ERCP	Involves creating G-G fistula	96%	~100%	17–22%	Up to 4	17 days	Yes
Common bile duct exploration		Allows for simultan-eous CCY	Risk of bile leak	90–95%	97%	7%	1	1 day	No
Spyglass		Single-operator system	Limited Availability	82–97%	93%	12–18%	1	1–4 days	No

## Data Availability

No new data were created or analyzed in this study. Data sharing is not applicable to this article.

## Abbreviations

The following abbreviations are used in this manuscript:

CBD	Common bile duct
ERCP	Endoscopic retrograde cholangiopancreatography
EDGE	EUS-directed transgastric access
EUS	Endoscopic ultrasound
LAMS	Lumen-opposing metal stent
LCBDE	Laparoscopic common bile duct exploration
PTC	Percutaneous transhepatic cholangioscopy
RNYGB	Roux-en-Y gastric bypass
